# Modulating Host Signaling Pathways to Promote Resistance to Infection by *Candida albicans*

**DOI:** 10.3389/fcimb.2017.00481

**Published:** 2017-11-21

**Authors:** Nick Carpino, Shamoon Naseem, David M. Frank, James B. Konopka

**Affiliations:** Department of Molecular Genetics and Microbiology, Stony Brook University, Stony Brook, NY, United States

**Keywords:** *Candida albicans*, Jnk1, Cbl-b, Sts-1, Sts-2

## Abstract

*Candida albicans* is a common human fungal pathogen capable of causing serious systemic infections that can progress to become lethal. Current therapeutic approaches have limited effectiveness, especially once a systemic infection is established, in part due to the lack of an effective immune response. Boosting the immune response to *C. albicans* has been the goal of immunotherapy, but it has to be done selectively to prevent deleterious hyperinflammation (sepsis). Although an efficient inflammatory response is necessary to fight infection, the typical response to *C. albicans* results in collateral damage to tissues thereby exacerbating the pathological effects of infection. For this reason, identifying specific ways of modulating the immune system holds promise for development of new improved therapeutic approaches. This review will focus on recent studies that provide insight using mutant strains of mice that are more resistant to bloodstream infection by *C. albicans*. These mice are deficient in signal transduction proteins including the Jnk1 MAP kinase, the Cbl-b E3 ubiquitin ligase, or the Sts phosphatases. Interestingly, the mutant mice display a different response to *C. albicans* that results in faster clearance of infection without hyper-inflammation and collateral damage. A common underlying theme between the resistant mouse strains is loss of negative regulatory proteins that are known to restrain activation of cell surface receptor-initiated signaling cascades. Understanding the cellular and molecular mechanisms that promote resistance to *C. albicans* in mice will help to identify new approaches for improving antifungal therapy.

## Introduction

The human fungal pathogen *Candida albicans* is commonly carried as a harmless commensal organism on the skin and mucosa. Under certain conditions, *C. albicans* can disseminate through the bloodstream to a wide range of tissues and progress to life-threatening systemic infections (Brown et al., 2012; Kullberg and Arendrup, 2015). Lethal infections commonly occur in immunocompromised patients. This pool of highly susceptible individuals is increasing due to advances in medical care for those whose immune system is suppressed by conditions that include cancer therapy, organ transplantation, and lymphoproliferative disorders (Pfaller and Diekema, 2010; Pfaller and Castanheira, 2016). However, serious infections also occur in other patient groups, such as those in the intensive care unit following abdominal or cardiac surgery (Das et al., 2011; Pfaller et al., 2012; Lortholary et al., 2014). Risk factors include the increased use of indwelling medical devices and catheters that provide sites for biofilm formation that can release a large inoculum. Surgical procedures that enable *C. albicans* to cross the skin and mucosal barriers are also a significant risk factor. Another contributing factor is the use of antibiotics against bacteria, which allows the endogenous commensal forms of *C. albicans* in the GI tract to overgrow and disseminate (Oever and Netea, 2014; Fan et al., 2015). Once established, systemic candidiasis is difficult to treat, as evidenced by a mortality rate of ~40% that has not decreased in spite of advances in antifungal therapy (Pfaller and Diekema, 2010; Das et al., 2011; Brown et al., 2012). Thus, new therapeutic strategies are needed to combat *C. albicans* infections (Rodrigues et al., 2016).

Modulating the immune system holds promise as a new therapeutic approach against systemic candidiasis. Innate immunity plays the key role in defense against *C. albicans*, although several branches of the immune system can contribute, as evidenced by the ability of experimental vaccines to promote resistance to *C. albicans* (Richardson and Moyes, 2015). In particular, neutrophils are thought to be critical, as neutropenic patients and animal models show greatly increased susceptibility to *C. albicans* (Romani et al., 1997; Gazendam et al., 2016). Therefore, one goal has been to use cytokine therapy to boost the numbers of innate immune cells and their responses to pathogenic fungi, especially in immunocompromised patients (van de Veerdonk et al., 2010; Ravikumar et al., 2015; Armstrong-James et al., 2017). For example, GM-CSF has been examined due to its ability to accelerate the proliferation and maturation of myeloid cells to produce more monocytes and neutrophils (Gadish et al., 1991; Bär et al., 2014; Kullberg et al., 2014). Another approach under investigation is to boost immune system function with factors such as Interferon gamma (van de Veerdonk et al., 2010; Ravikumar et al., 2015). In spite of some promising reports, the results for these types of immunotherapy approaches have been controversial (van de Veerdonk et al., 2010; Ravikumar et al., 2015). Furthermore, an underlying concern with the use of cytokine therapy is the potential to induce a hyper-inflammatory state that would cause deleterious collateral damage to the host (Safdar, 2007; van de Veerdonk et al., 2010).

Recent studies in mice indicate there may be more selective ways to optimize the ability of the immune system to kill *C. albicans* without causing collateral damage to the host from inflammation. Three different types of mutations have been identified that make mice more resistant to *Candida* infection (Naseem et al., 2015; Wirnsberger et al., 2016; Xiao et al., 2016; Zhao et al., 2017). Significantly, all three types of mutant mice are resistant to infection without causing hyper-inflammatory responses and tissue destruction. A common underlying theme is that all three mutations affect genes involved in cell signaling pathways; two of the genes have been shown to regulate signal transduction downstream of C-type lectin receptor-mediated pathogen recognition. One class of mutations involved deletion of a pair of related genes, Sts-1 and Sts-2, that act as phosphatases (Naseem et al., 2015). Interestingly, the Sts phosphatases have a structure distinct from typical protein phosphatases (Mikhailik et al., 2007). Another mutation affected the Cbl-b E3 ubiquitin ligase, which regulates cell signaling by controlling ubiquitination of proteins (Wirnsberger et al., 2016; Xiao et al., 2016). The third type of mutation affected Jnk1, which is a member of the family of MAP kinases (Zhao et al., 2017). For the latter two strains, it has been shown that the resistance can be transferred via bone marrow transplant, confirming that the improved antifungal activity is due to changes in the hematopoietic system. These resistant mice are now being analyzed to identify ways to optimize the immune response to fungal infections. The focus of this review will therefore be on comparing mouse mutations that increase or decrease susceptibility to *C. albicans* infection as a way to identify novel therapeutic strategies.

## Factors that increase susceptibility to *Candida* infection

### Susceptibility of humans to *C. albicans*

When *C. albicans* crosses the skin or mucosal barriers via medical procedures or injury, it can lead to virulent systemic infections even in healthy adults. Much of what is known about anti-*Candida* host responses in humans comes from analysis of immunocompromised patients. Studies on different patient populations indicate that innate immunity, especially neutrophils, is important for resistance to *C. albicans* (Pfaller and Diekema, 2010; Duggan et al., 2015; Pfaller and Castanheira, 2016; Lionakis et al., 2017). Patients that are neutropenic due to hematological diseases, such as leukemia, or as a consequence of therapy for cancer or organ transplantation are more susceptible to fungal infections (Nesher and Rolston, 2014; Teoh and Pavelka, 2016). Interestingly, the immune response to oral candidiasis differs from hematogenously-disseminated candidiasis. T cells, particularly the Th17 subtype, are critical for preventing oral candidiasis but not for systemic candidiasis (Whibley and Gaffen, 2014; Salvatori et al., 2016). This is supported by the fact that AIDS patients are quick to develop oral candidiasis as T cell counts decrease, but do not quickly progress to systemic infections (Cassone and Cauda, 2012).

Genetic studies have identified specific immune functions that are important for resistance to systemic candidiasis. For example, patients with chronic granulomatous disease (CGD) display increased susceptibility to *C. albicans* and other fungi due to mutations that affect the nicotinamide adenine dinucleotide phosphate (NADPH) oxidase complex. These mutations lead to defects in production of reactive oxygen species that are used to attack pathogens, and consequently decreased ability to kill *C. albicans* (Smeekens et al., 2013b; Wang et al., 2016). Other genetic disorders, such as severe congenital neutropenia and leukocyte adhesion deficiency type 1, confirm the importance of appropriate levels of phagocytic cells and their ability to migrate to the site of infection in preventing invasive candidiasis (Smeekens et al., 2013b; Wang et al., 2016). Myeloperoxidase deficiency is also linked to increased susceptibility to infection (Antachopoulos et al., 2007). However, the rate of *C. albicans* infections in these patients is low, suggesting that other aspects of host defense may play a major role (Winkelstein et al., 2000; Pradhan et al., 2017). Genome sequences of patient populations are also revealing DNA polymorphisms associated with increased susceptibility to *C. albicans* in humans that indicate a role for genes encoding pattern recognition receptors, cytokines, and signaling pathways including VAV3, TAGAP, CD58, CXCR1, type I interferons, and CX3CR1(Lionakis et al., 2013; Smeekens et al., 2013a; Kumar et al., 2014; Roth et al., 2016; Swamydas et al., 2016). However, these latter mutations may be part of multigenic effects on susceptibility to infection (Wang et al., 2016).

### Susceptibility of mice to *C. albicans*

Mice are the most common animal model for systemic candidiasis. One reason is that the availability of a wide range of mutant mouse strains has permitted a genetic dissection of host factors that mediate the response to infection. Another reason is that mice are a good model for studying many aspects of candidiasis in humans. Infection is typically initiated by injection of *C. albicans* into the bloodstream via the tail vein, followed by hematogenously disseminated candidiasis and death from progressive sepsis, as indicated by worsening hypotension, tachycardia, hypothermia, metabolic acidosis, and renal insufficiency (Spellberg et al., 2005; Hohl, 2014; Duggan et al., 2015). This resembles the progression of sepsis seen during severe clinical cases of *C. albicans* infections. One difference, however, is that the kidney is the main site of *C. albicans* replication in mice, whereas this fungus can infect a variety of organs in humans (Spellberg et al., 2005; Kullberg and Arendrup, 2015). Also, although mice do not normally carry *C. albicans* as a commensal organism in the G.I. tract, they can be colonized after antibiotic treatment (Koh et al., 2008).

Wild type mice that are immune competent are susceptible to *C. albicans*, but require a correspondingly higher dose to cause a severe systemic infection than do immunocompromised mice. The analysis of targeted mutations in mice has demonstrated a key role for innate immunity, similar to humans, including a critical role for neutrophils (Netea et al., 2008; Miramon et al., 2013; Lionakis et al., 2017), inflammatory monocytes (Ngo et al., 2014; Domínguez-Andrés et al., 2017), and resident macrophages (Lionakis et al., 2013). In addition to the analysis of mice with targeted gene knockout mutations, the screening of different inbred mouse strains revealed big differences in susceptibility to *C. albicans* infection (Peltz et al., 2011; Radovanovic et al., 2011). Interestingly, mouse strains that were highly susceptible to *C. albicans* contained mutations in the complement pathway. Mutation of C5 was implicated in causing the greatly increased susceptibility of A/J mice to *C. albicans* infection, and this was confirmed by genetic crosses between susceptible A/J and resistant C57BL/6J mouse strains (Mullick et al., 2006). Mapping of susceptibility determinants in 16 inbred mouse strains revealed genetic interactions between complement C5 and C1r/s alleles that were predictive of susceptibility to *C. albicans* infection (Peltz et al., 2011).

The mouse model for candidiasis has also permitted the identification of *C. albicans* virulence factors that promote the pathogenesis. One underlying virulence factor is the ability of *C. albicans* to transition from growing as spherical budding cells and to instead form long chains of filamentous hyphal cells that grow invasively into tissues (Sudbery, 2011). The ability to switch to filamentous growth is also important for *C. albicans* to form biofilms on catheters and other medical devices (Finkel and Mitchell, 2011). Cells induced to form hyphae also show increased expression of virulence factors including adhesin proteins that promote biofilm formation, enzymes that protect the cells from oxidative attack by the immune system, and Candidalysin, a secreted peptide that damages epithelial cell membranes (Gleason et al., 2014; Moyes et al., 2016; Woolford et al., 2016).

## Host inflammatory responses exacerbate damage due to infection by *C. albicans*

Studies in mice and humans indicate that the inflammatory response to *C. albicans* infection causes significant collateral damage to the host (Duggan et al., 2015). Thus, although the host immune response is important for fighting *C. albicans* infections, the inflammatory response also exacerbates the effects of infection. For example, improved survival to bloodstream infection by *C. albicans* was observed in mice lacking the TEC tyrosine kinase (Zwolanek et al., 2014). The *Tec1*−*/*− mice were defective in stimulating Caspase 8 to process and activate IL1-β, leading to a reduced inflammatory response. However, the extent of fungal growth in the kidneys of *Tec1*−*/*− mice was similar to the wild type mice. This indicates that although the *Tec1*−*/*− mice showed less inflammatory damage, these mice were not more efficient at killing *C. albicans*. A similar effect was also observed when neutrophil recruitment to the kidney was impaired due to mutation of the CCR1 chemokine receptor (Lionakis et al., 2012) or mutation of IL-17C (Huang et al., 2016). Mice lacking the receptor for type I interferons (*Ifnar1*−*/*−) were also found to survive a low dose of *C. albicans* infection longer than wild type control mice due to reduced renal immunopathology (Majer et al., 2012). Improved survival could also be observed after pharmacological inhibition of the inflammatory response in wild type cells (Majer et al., 2012). However, these results are controversial because del Fresno et al found that *Ifnar1*−*/*− mice were more susceptible to infection by *C. albicans* (del Fresno et al., 2013). Altogether, these results are analogous to observations for other types of microbial pathogens wherein the pathology due to infection is often exacerbated by the host inflammatory response (Casadevall and Pirofski, 2003; Pirofski and Casadevall, 2008).

Other mutant strains of mice that are resistant to *C. albicans*, but show deleterious effects due in part to hyper-inflammation have been reported. For example, mice lacking the CD37 tetraspanin were resistant to *C. albicans* and had a lower fungal burden in the kidney (van Spriel et al., 2009). However, cd37−/− mice showed significantly increased levels of serum IgA and proinflammatory IL-6 by day 3 postinfection, both of which were concluded to aid in the clearance of *C. albicans*. CD37 associates with Dectin 1, and its mutation results in improved signaling to increase IL-6 production (Meyer-Wentrup et al., 2007). In addition, elevated serum IgA levels in the absence of CD37 led to glomerular IgA deposition and increased levels of inflammatory phagocytes within the kidney, making cd37−/− mice prone to spontaneous nephropathy (Rops et al., 2010). Another example is that MCPIP1+/− heterozygous mice (also known as Zc3h12a+/−) lacking one copy of this endoribonuclease gene were also more resistant to *C. albicans* (Garg et al., 2015). MCPIP1 is a negative regulator of IL-17, so the loss of one copy of this gene resulted in elevated IL-17 signaling and improved ability to resist *C. albicans* (although the fungal burden in the kidney was not assessed). However, the MCPIP1+/− mice also showed some increased levels of inflammation and displayed increased pathology in experimental autoimmune encephalomyelitis and in pulmonary inflammation (Garg et al., 2015), and a complete deletion of MCPIP1 causes a lethal inflammatory disease (Miao et al., 2013).

## Mutant mice that display resistance to *C. albicans* without hyper-inflammation

Immune activation and inflammation are important for resisting *C. albicans*, but as discussed above, it can be difficult to enhance fungal killing activity without deleterious collateral damage to the host due to hyper-inflammation or other side effects. However, recent studies have identified mutant mouse strains that are more effective at resisting *C. albicans* infection without significant deleterious effects. As will be described in more detail below, these mice have defects in genes encoding the cell signaling proteins Cbl-b, Jnk1, and Sts-1 and −2.

### *Cbl-b*−/− mice

**Figure 1:**
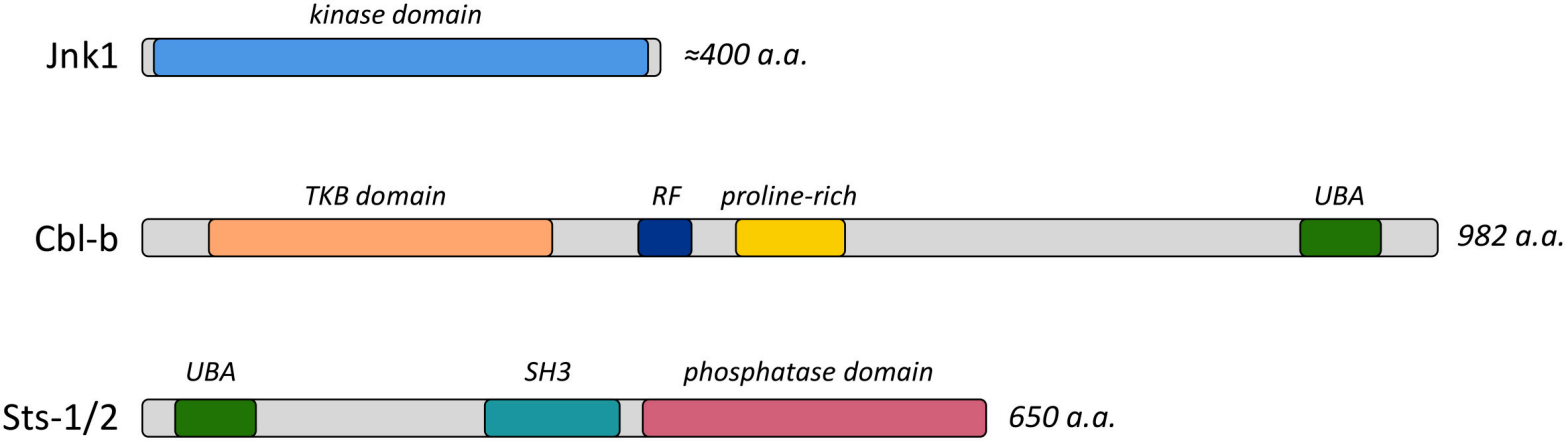
Functional domains of Jnk1, Cbl-b, and Sts-1/2 proteins. Diagrams indicate the important functional domains and the length of the proteins. Abbreviations include TKB, Tyrosine Kinase Binding; RF, Ring Finger; UBA, Ubiquitin association; and SH3, Src homology-3.

Cbl-b is one of three homologous proteins of the Cbl ubiquitin ligase enzyme family (Liu, 2004), all of which possess a modular structure with diverse protein interaction motifs and a RING finger ubiquitin ligase domain (Figure 1). It is highly expressed within the immune system, and has been implicated in regulating signaling pathways downstream of surface receptors localized on both innate and adaptive immune cells (Liu et al., 2014). Cbl-b regulates diverse signaling cascades by targeting receptors and downstream effector molecules for ubiquitin-mediated internalization and/or degradation (Thien and Langdon, 2005). In addition, it functions as an adaptor protein in a ligase-independent fashion (Guo et al., 2012). Recent studies evaluated the role of Cbl-b in the immune response to *C. albicans* infection by examining the responses of *Cbl-b*−*/*− mice (Wirnsberger et al., 2016; Xiao et al., 2016; Zhu et al., 2016). *Cbl-b*−*/*− mice are highly resistant to disseminated candidiasis, with resistance characterized by reduced weight loss following infection, an absence of inflammatory damage, and enhanced survival. Within 24–48 h of bloodstream infection *Cbl-b*−*/*− mice show signs of enhanced fungal clearance, as illustrated by a 10-fold reduction in fungal burden within different peripheral organs.

**Figure 2:**
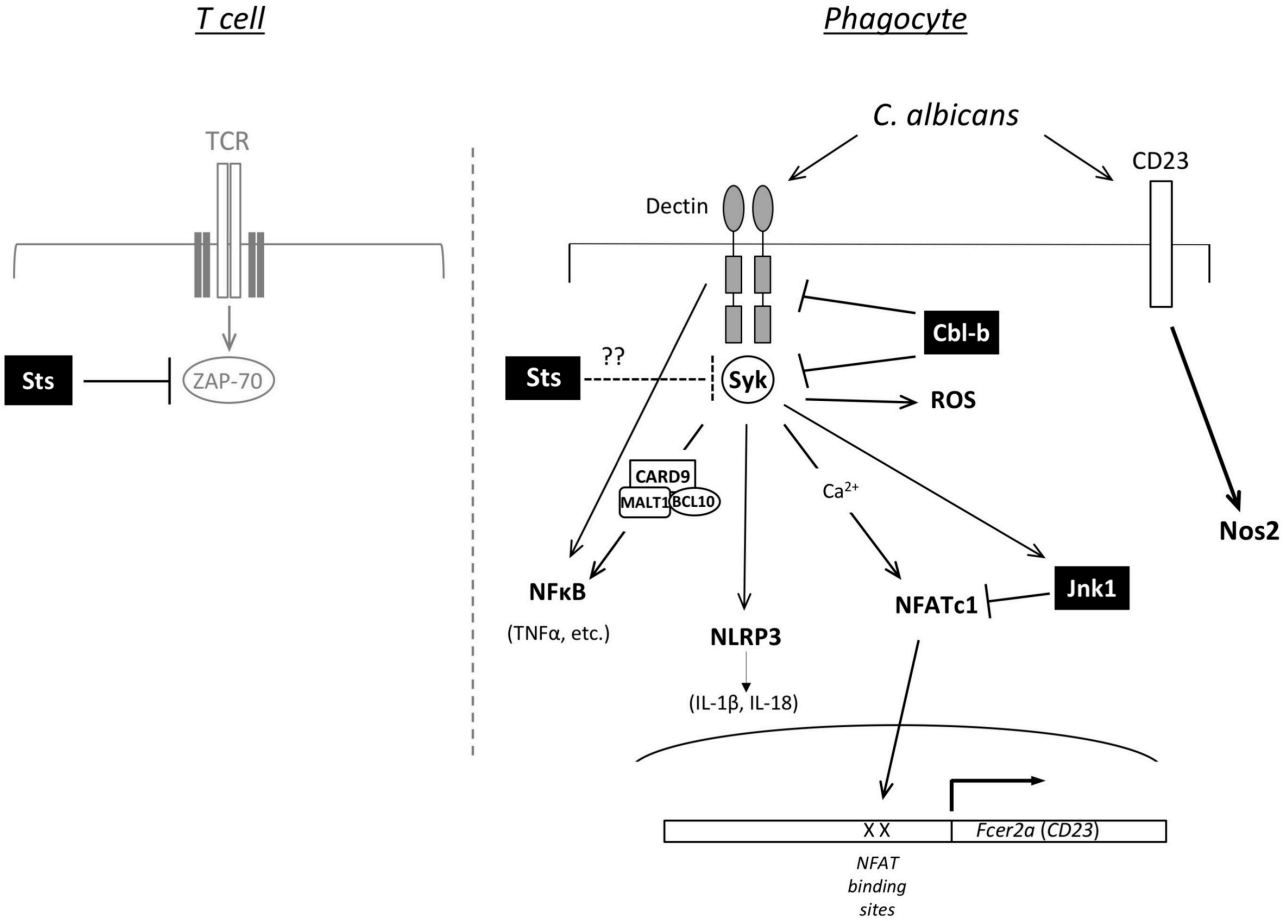
Predicted functions of Jnk1, Cbl-b, and Sts-1/2 in cell signaling. Model for how loss of function of Jnk1, Cbl-b, or the Sts proteins promotes resistance to *C. albicans*. The right side shows a diagram of signaling pathways in phagocytic cells of the innate immune system. Jnk1 negatively regulates the NFATc1 transcription factor to suppress expression of Fcer2a (CD23). CD23 activation induces expression of Nos2 (iNOS). Cbl-b is an E3 ligase that regulates the ubiquitination of Dectin receptors (Dectin-1,−2,−3) and the Syk protein kinase, thereby regulating their levels of expression. In T cells Sts-1 and −2 are phosphatases that counteract the activation of the kinase Zap-70 downstream of TCR engagement (left side). Syk is a homolog of Zap-70 expressed in phagocytes, suggesting the Sts proteins could negatively regulate Syk activity.

*Cbl-b*−*/*− bone marrow-derived myeloid mononuclear phagocytes, but not neutrophils, display molecular and functional alterations that could underlie enhanced fungal restriction. In particular, surface expression of Dectin-1 receptors on innate immune cells that sense β-glucan in fungal cell wall was stabilized following interactions between fungal cells and bone marrow derived macrophages (Xiao et al., 2016). As a result of reduced ligand-mediated receptor internalization and degradation in the absence of Cbl-b, expression of Dectin receptors are maintained at higher levels at the cell surface where they sense *C. albicans* (Zhu et al., 2016). In addition, the downstream kinase Syk is hyper-activated following stimulation of *Cbl-b*−*/*− phagocytes (Wirnsberger et al., 2016; Xiao et al., 2016). Thus, Dectin-1,−2, and −3 along with Syk appear to be targets of Cbl-b mediated ubiquitination and down-modulation, with downstream signaling pathways and effector functions potentiated in the absence of Cbl-b expression (Figure 2). Among the downstream effects of Cbl-b deletion are increased production of pro-inflammatory cytokines and increased ROS production following stimulation of phagocytes with *C. albicans* and other Dectin receptor agonists. Interestingly, *in vivo* depletion of phagocytes by chlodronate administration prior to infection abrogates the protective effects conferred by Cbl-b deficiency, highlighting their important role (Wirnsberger et al., 2016). These data are consistent with a model in which heightened anti-fungal effector responses of key phagocyte populations support a more rapid and effective elimination of the fungus, and thereby decreased inflammatory damage during the course of infection.

### *Jnk1*−/− mice

Jnk1 is a member of the MAPK family of enzymes (Figure 1), an ancient and evolutionarily conserved family of kinases that are critical in many cell types for transducing extracellular signals (Davis, 2000; Dong et al., 2002). Jnk1 has complex and pleiotropic functions in a variety of physiological responses and pathological conditions, mainly due to its role in activating or inhibiting diverse transcription factors (Zeke et al., 2016). Interestingly, a recent study by Zhao et al. found that mice lacking Jnk1 expression are more resistant to bloodstream *C. albicans* infection than wild type mice (Zhao et al., 2017). As with *Cbl-b*−*/*− mice, the resistance of *Jnk1*−*/*− mice is characterized by reduced inflammatory damage within the kidney, reduced kidney fungal CFUs 48 h after infection, and increased survival. Furthermore, enhanced activation of phagocytes such as macrophages and dendritic cells, but not neutrophils, also appears to be responsible for the protection evident in *Jnk1*−*/*− mice. This effect appears to be specific to Jnk1, as inhibition of the ERK MAPK causes the opposite effect—increased susceptibility to *C albicans* infection (Jia et al., 2014).

Despite similarities between the responses of the two mutant strains to *C. albicans* infection, the underlying molecular mechanisms by which *Jnk1*−*/*− animals resist infection appear to be substantially different from the mechanisms underlying the resistance of *Cbl-b*−*/*− mice. For example, in contrast to the bone marrow derived macrophages (BMDMs) from *Cbl-b*−*/*− mice, Jnk1−/− BMDMs do not produce elevated levels of pro-inflammatory cytokines following stimulation with live fungal cells or fungal cell wall components. Rather, Jnk1 deficiency leads to up-regulation of the C-type lectin receptor Fcer2a (CD23) (Zhao et al., 2017). This is likely the result of increased activation of NFATc1, an upstream transcription factor that is normally suppressed by Jnk1. Increased nuclear localization and binding of NFATc1 to the CD23 promoter, increased up-regulation of CD23 gene expression, and increased levels of CD23 receptor expression can all be detected in *Jnk1*−*/*− macrophages (Zhao et al., 2017). As a secondary consequence of increased CD23 expression, phagocytes lacking Jnk1 display elevated levels of iNOS (Nos2) expression and fungal-induced NO production (Figure 2). They also produced moderately elevated levels of ROS. Because reactive oxygen/nitrogen species have critical anti-microbial killing properties, Jnk1 deficiency thereby appears to endow phagocytes with increased fungicidal capabilities. Thus, a biochemical feed-forward loop that involves increased NFATc1 activation, up-regulation of fungal receptor Fcer2a (CD23), and consequent increased Nos2 enzyme levels and NO production better enables the innate immune response to overcome a highly virulent fungal pathogen.

### *Sts*−/− mice

Another mouse strain that was recently shown to resist *C. albicans* was created by disruption of the Sts-1 and Sts-2 genes (Carpino et al., 2004; Naseem et al., 2015). The Sts proteins are homologous protein phosphatases that share over-lapping functions (Mikhailik et al., 2007; Tsygankov, 2013). They have a distinct tripartite structure consisting of two protein-interaction domains (UBA and SH3) and a C-terminal 2H-phosphatase domain (Figure 1) that is structurally and enzymatically very distinct from intracellular protein tyrosine phosphatases that are more commonly known to regulate immune signaling pathways (Mikhailik et al., 2007; Rigden, 2008). To date, the Sts proteins have been established as negative regulators of diverse signaling pathways, most prominently within cells of the mammalian immune system. For example, within T cells, the Sts enzymes contribute to setting the threshold of T cell activation by targeting the important kinase Zap-70 downstream of the T cell receptor (Carpino et al., 2004; San Luis et al., 2011). Additionally, Sts-1 has been shown to control signaling downstream of both GPVI-FcRγ in platelets and FcϵR in mast cells by targeting Syk, a Zap-70 homolog (de Castro et al., 2012; Reppschläger et al., 2016).

*Sts*−*/*− mice are profoundly resistant to systemic candidiasis caused by bloodstream *C. albicans* infection, similar to *Cbl-b*−*/*− and *Jnk1*−*/*− mice. Along with enhanced survival, *Sts*−*/*− animals displayed a significant reduction in kidney fungal burden by 24 h postinfection, sharply diminished levels of many inflammatory molecules, and an absence of inflammatory lesions within the kidney (Naseem et al., 2015). While the cellular and molecular basis for the enhanced resistance of *Sts*−*/*− mice has not been defined, it is possible that the Sts proteins regulate the threshold of activation of bone marrow derived cells that play a critical role in the anti-*Candida* immune response (Figure 2). Analogous to the heightened activation seen in T cells lacking Sts expression, one likely scenario is that innate immune cells lacking Sts expression respond to microbial challenge more actively and effectively, allowing them to overcome infection more readily. Support for this model comes from the recent observation that *Sts*−*/*− monocytes restrict the intracellular-replicating bacterial pathogen *Francisella tularensis* LVS with 10-fold greater efficiency than wild type monocytes, thereby enhancing host survival following a lethal infectious dose (Parashar et al., 2017). It remains to be determined whether the same mechanism responsible for enhanced *Francisella* clearance is also responsible for optimal fungal clearance during *C. albicans* systemic infection. However, as with *Cbl-b*−*/*− and *Jnk1*−*/*− mice, the resistance of *Sts*−*/*− mice supports the idea that potentiation of cellular anti-fungal effector mechanisms by inactivation of negative regulatory mechanisms can have profound consequences on the outcome of host-pathogen interactions.

## Summary

To date, three different types of mutations have been identified that make mice more resistant to *C. albicans* without causing hyper-inflammation, namely *Cbl-b*−*/*−, *Jnk1*−*/*−, and *Sts*−*/*− animals (Naseem et al., 2015; Wirnsberger et al., 2016; Xiao et al., 2016; Zhao et al., 2017). All of the corresponding genes are thought to play a role in negatively regulating different cell signaling pathways (Figure 2). The Jnk1 kinase acts to negatively regulate the NFATc1 transcription factor and Cbl-b is an E3 ligase that regulates the stability of Dectin receptors. The role of Sts-1 and −2 in promoting resistance is likely due to their ability to counteract protein kinases by acting as phosphatases. Thus, it is consistent that loss of function mutations for all three of these negative regulators leads to up-regulation of functions that promote the killing of *C. albicans*.

The fact that loss of function of Jnk1, Cbl-b or the Sts proteins promotes resistance to *C. albicans* suggests that they are good candidates as targets for drugs to inactivate their function. Optimizing the immune response to avoid collateral damage in ways identified by the mutant mice has potential for new therapeutic approaches. Boosting an effective response to *C. albicans* could also have efficacy in improving other therapeutic approaches. For example, it could synergize with the killing activity of current antifungal drugs or could improve the efficacy of vaccines by lowering the fungal burden. Therapeutic intervention by targeting Jnk1 and Cbl-b has been validated in mouse studies. For example, administration of the Jnk1 inhibitors SP600125 or JNK-IN-8 leads to increased resistance to bloodstream *C. albicans* infection (Zhao et al., 2017). Interference of Cbl-b function via systemic *in vivo* delivery of Cbl-b siRNA or treatment with a cell permeable peptide that blocks Dectin-Cbl-b interactions also promoted enhanced anti-fungal immunity and survival (Wirnsberger et al., 2016; Xiao et al., 2016).

The lessons learned from studies with mice that are resistant to *C. albicans* will likely have significance for optimizing the immune response to other pathogens. In this regard it is interesting that *Sts*−*/*− mice are also resistant to infection by the bacterial pathogen *Franciscella tularensis* (Parashar et al., 2017). Finding new ways to selectively boost immune function will be particularly important for types of infections that are characterized by excessive inflammatory damage. For example, it will be interesting to test new immunotherapy strategies in cases of fungal *Cryptococcal* meningitis (Chen et al., 2013) and bacterial meningitis (Brouwer et al., 2015). These types of meningitis are often lethal due to susceptibility of the brain to damage from inflammatory responses (Panackal et al., 2016). Immunosuppressive drugs have been used for treatment of certain types of meningitis to minimize inflammatory damage even though this also blocks the ability of the immune system to fight infection (Chen et al., 2013; Brouwer et al., 2015). Thus, discovering new ways to optimize the immune response to *C. albicans* without deleterious inflammatory damage to the host will provide new insights for identifying novel therapeutic approaches for other pathogens.

## Author contributions

NC, SN, DF, and JK conceived of the work. NC and JK drafted the manuscript, and then SN and DF were responsible for revising it.

### Conflict of interest statement

The authors declare that the research was conducted in the absence of any commercial or financial relationships that could be construed as a potential conflict of interest.
